# Comparison of cases with and without acute liver injury in pregnant women with SARS-CoV-2 infection; obstetric and neonatal outcomes

**DOI:** 10.12669/pjms.40.3.7746

**Published:** 2024

**Authors:** Nur Gozde Kulhan, Gokcen Orgul, Fazil Avci, Mehmet Kulhan

**Affiliations:** 1 Nur Gozde Kulhan Department of Gynaecology and Obstetrics, Konya City Hospital, Konya, Turkey; 2 Gokcen Orgul, Selcuk University Medical Faculty, Gynaecology and Obstetrics Department, Konya, Turkey; 3 Fazil Avci, Selcuk University Medical Faculty, Gynaecology and Obstetrics Department, Konya, Turkey; 4 Mehmet Kulhan, Selcuk University Medical Faculty, Gynaecology and Obstetrics Department, Konya, Turkey

**Keywords:** COVID-19, Liver damage, Pregnancy, SARS-CoV-2

## Abstract

**Background and objective:**

The effect of SARS-CoV-2 infection on the liver during pregnancy and the impact of SARS-COV-2-related liver injury during pregnancy on obstetric and neonatal outcomes are not yet clear. The aim of this study was to determine the clinical features of pregnant women at risk of liver injury and to investigate the effect of liver dysfunction on obstetric and perinatal outcomes.

**Methodology:**

Pregnant women who were followed up and treated at Selcuk University Medical Faculty Hospital and diagnosed with COVID-19 were determined retrospectively. All pregnant women whose PCR test results were positive between March 1, 2020 and July 31, 2022 were included. A total of 96 PCR positive pregnant women were included in the study. The patients were divided into two groups as those with and without liver damage. Both groups were compared in terms of obstetric and neonatal outcomes.

**Results:**

While liver damage findings were observed in 34.4% of the 96 pregnant included in the study; No liver damage was observed in 65.6% of the patients. White blood cell, neutrophil, ferritin, D-dimer, troponin, C-reactive protein, systemic immune-inflammation index, interleukin-6, alanine aminotransferase, aspartate aminotransferase and lactate dehydrogenase levels were higher in patients with liver injury compared to pregnant women without liver injury. Prematurity, premature rupture of membranes, preterm premature rupture of membranes, preeclampsia and fetal death were observed relatively more in the patient group with liver injury, there was no statistical significiant difference between the groups in terms of these complications. Unfortunately, maternal death occurred in four mothers with liver injury and in one patient without liver injury. Birthweight, APGAR scores and obstetric complication rates were similar between two groups.

**Conclusion:**

Our study showed that pregnant patients with liver damage had worse inflammatory response than those without liver damage. Women with elevated liver enzymes tend to have severe disease, but obstetric and perinatal outcomes were similar between groups with and without liver damage.

## INTRODUCTION

Severe Acute Respiratory Syndrome Coronavirus-2 (SARS-CoV-2) primarily affects the respiratory system. However, recent data revealed that this infection is a systemic infection with multiorgan involvement including liver, pancreas, heart and kidney with or without clinical findings. About half of people infected with SARS-CoV-2 have liver dysfunction that becomes more aggravated as the severity of the disease.^1^,^2^ SARS-COV-2 reaches the liver through the high number of Angiotensin Converting Enzyme-2 (ACE2) receptors on the hepatic bile duct cell surface and may cause abnormalities in liver function tests.^3^-^5^ There are also publications showing that viruses that affect the respiratory system can also damage liver cells through the CD8+ mediated immune response.^6^

Although a relationship between SARS-CoV-2 and abnormal liver function tests has been suggested,^7^,^8^ evidence from recent epidemiological and clinical studies of abnormal liver function in patients with SARS CoV-2 infection is largely inconsistent and contradictory.^9^-^11^ The effect of the SARS CoV-2 infection occurring during pregnancy on the liver is clearly unknown. There are only two studies on this subject. According to the results of these studies, the elevation of liver enzymes varies between 23-44%. It was concluded that pregnant women with liver damage had worse inflammation than those without liver damage. It was concluded that liver function tests should be monitored in all pregnant following SARS CoV-2 diagnosis.^12^-^14^ Evidence-based information showing that pregnant women are more susceptible to SARS-COV-2 has not yet been reported.

The effect of liver damage associated with SARS-COV-2 in pregnancy on obstetric and neonatal outcomes is not yet clear.^15^-^17^ There is no study in the literature examining the effect of liver dysfunction due to SARS-COV-2 on obstetric and neonatal outcomes. The present study will be the first study to examine this issue. In this study, the data of cases with and without liver damage in pregnant women whose diagnosis of Coronavirus disease 2019 (COVID-19) confirmed by polymerase chain reaction PCR test were compared retrospectively. The aim of this study was to determine the clinical features of pregnant women at risk for liver damage and to investigate the effect of liver dysfunction on obstetric and perinatal outcomes.

## METHODS

Pregnant women who were followed up and treated at Selcuk University Medical Faculty Hospital and diagnosed with COVID-19 were determined retrospectively. All pregnant women whose PCR test results were positive between March 1, 2020 and July 31, 2022 were included in the study. The clinical, sociodemographic and obstetric data of the patients were obtained from the hospital computer system together with patient files.

A total of 96 PCR positive pregnant women were included in the study. The patients were divided into two groups as those with and without liver damage. The group with liver damage consisted of 33 patients and the group without liver damage consisted of 63 patients. Liver injury was defined as an increase in any of the following parameters: alanine aminotransferase (ALT) >40 U/L, aspartate aminotransferase (AST) >40 U/L, and total bilirubin (TBIL) >17.1 lmol/L 1. Patients with elevation in any of these values were assigned to the group with liver damage. Patients with normal liver function tests constituted into the control group. Demographic data of patients age, gender, comorbidities, symptoms, laboratory data test results at the first admission and hospitalization in terms of AST, ALT, ALP (alkaline phosphatase), GGT (gamma glutamyl transferase), total bilirubin, albumin, leukocyte, neutrophil, lymphocyte, platelet, creatinine, lactate dehydrogenase (LDH), prothrombin time (PT), activated thromboplastin time (aPTT), C-reactive protein (CRP), procalcitonin, D-dimer, ferritin, drugs used in service and intensive care treatment recorded retrospectively.

The neutrophil-lymphocyte ratio, platelet-lymphocyte ratio, and prognostic index based on systemic immune-inflammation index (SII) were calculated from the complete blood count at admission.^18^ These parameters have been shown to correlate with the severity of COVID-19.^19^ Elevated liver function tests were investigated on the first day of hospitalization and follow-up of patients with positive SARS-CoV-2 test. Both groups were compared in terms of disease severity, laboratory findings, comorbidities, obstetric history, symptoms, length of hospital stay, time to delivery, mode of delivery, and perinatal outcomes. Less than 18 years of age, liver cirrhosis, chronic liver disease, pancreatitis and cholangitis were accepted as exclusion criteria.

### Statistical analysis

All analyzes were performed using IBM SPSS 20 software. Comparisons in demographic variables between both groups were made with independent sample t-test or Mann-Whitney U test for continuous variable and chi-square test for categorical variables. P-values were presented and statistical significance was accepted as p< 0.05.

### Ethical approval

This study was approved by the Ethics Committee of the Faculty of Medicine of our University with the date of 23.08.2022 and Decision No: 2022/359. Informed consent was obtained from all individual participants included in the study before treatment.

## RESULTS

A total of 96 pregnant patients were included in this study, while laboratory findings consistent with liver injury were observed in 33 34.4% patients; any of AST, ALT and TBIL levels were within the normal range in 63 65.6% patients. The mean age of the patients with liver injury was 32.03±4.80 years, two of the patients with liver injury had chronic hypertension and three had asthma and hypothyroidism as comorbid diseases. The mean age of patients without liver injury was 30.31±5 years, and patients in this group had one diabetes mellitus, two hypertension and seven other comorbid diseases. Cough, dispne, fatigue-weakness and muscle-joint pain were the most common initial symptoms in both groups.

All of these symptoms were observed less frequently in pregnant women with liver injury. White blood cell (WBC), Neutrophil, Ferritin, D-dimer, Troponin, CRP, SII, interleukin-6 IL-6, AST, ALT and LDH levels were higher in patients with liver injury compared to pregnant women without liver injury. Demographic and laboratory data are presented in Table-I. There was no statistical difference between pregnant patients with and without liver injury in terms of time from COVID-19 onset to hospitalization and obstetric treatment. While 78.8% of 33 pregnant women with liver injury gave birth by cesarean section, the CS rate was 74.6% in the group without liver injury. There was no difference between the two groups in terms of delivery types p >0.05. Although prematurity, Premature Rupture of Membranes PROM, Preterm premature rupture of membranes (PPROM), preeclampsia and fetal death were observed relatively more in the patient group with liver injury, there was no statistically significant difference between the groups in terms of these complications. Unfortunately, maternal death occurred in four mothers with liver injury and in one patient without liver injury.

**Table-I:** Charasteristics of patients diagnosed with COVID 19.

Variables		Control group n=63	Patient group n=33	P value
Age, year		30,31±5,00	32,03±4,80	0,130
Gravida, n		3,0±1,41	3,20±1,33	0,408
Parity, n		1,50±1,12	1,84±1,15	0,145
Contact history		4 6,3 %	1 3,0 %	0,437
Complaint		53 84,1 %	30 91,0 %	0,278
	Fever	8 12,7 %	7 21,2 %	0,211
	Chill-Shiver	0	2 6,1 %	0,116
	Cough	36 57,1 %	25 75,6 %	0,005
	Dispne	22 35,0 %	20 60,6 %	0,014
	Tiredness-Fatigue	24 38,1 %	17 51,5 %	0,148
	Sore throat	9 14,3 %	3 9,1 %	0,352
	Taste disorder	5 8,0 %	2 6,1 %	0,546
	Olfactory-Sensory Loss	4 6,3 %	1 3,0%	0,437
	Stomach ache	1 1,66 %	3 9,1 %	0,116
	Nausea-Vomiting	2 3,2 %	3 91, %	0,220
	Muscle Joint Pain	21 33,3%	13 39,4 %	0,355
Coexistence disease				0,791
	Diabetes mellitus	1	0	
	Hypertension	2	2	
	Other	7	3	
Laboratory parameters				
	WBC x10^9^/L	8,24±3,4	9,4±2,9	0,050
	Neutrophils x10^9^/L	6,4±3,1	7,8±2,9	0,014
	Lymphocytes x10^9^/L	1,2±0,6	1,0±0,5	0,136
	NLR	5,25 1,0-32	8,1 1,1-38,5	0,122
	Platelets x10^9^/L	204,97±60,11	230,822±91,18	0,361
	PT sec	10,8 9,3-13,2	10,1 9,2-20,6	0,009
	APTT sec	27,7±5,0	28,5±3,9	0,490
	INR sec	0,9 0,8-1,4	0,8 0,8-1,7	0,015
	PCT ng/ml	0,05 0- 110	0,23 0,02-100	0,699
	AST U/Ll	22,0 10,0-38,0	73,0 22,0-1504,0	0,001
	LDH	213,0 131,0-450,0	349,0 204,0-1780,0	0,001
	ALT U/L	13,5 1,0-31,0	54,0 14,0-1008,0	0,001
	Albümin	3,5 2,0-4,70	3,1 2,1-5,7	0,029
	Ferritin	18,2 4,0-974,0	104,5 9,8-2253,0	0,001
	D dimer	685,0 178,0-4200,0	1159,0 147,0-8912,0	0,032
	Troponin	3,4 2,1-48,7	4,5 2,3-49,5	0,049
	Total Bilirubin µmol/L	0,5 0,1-3,3	0,6 0,2-54,0	0,152
	BUN mmol/L	14,6±4,7	12,8±4,7	0,076
	Cr	0,5±0,1	0,87±1,8	0,116
	CRP mg/L	19,7 1,75-254,0	78,7 5,7-227,0	0,001
	IL-6 pg/ml	6,1 0,9-127,9	26,0 2,2-258,2	0,001
	SII	1026,0 3,6-6048	1578,6 466,2-8816,5	0,007
	Fibrinogen	462,5±112,9	496,1±116,0	0,148
Blood gas results				
	pH	7,39±0,03	7,4±0,02	0,700
	laktat	1,5±0,7	1,4±0,7	0,764
	pO2	94,0±5,6	92,7±9,7	0,233
	pCO2	36,3 24,3-46,1	34,8 25,3-42,6	0,130
	sO2	96,1 45,4-99,2	96 36,6-98,5	0,835

**WBC:** White blood count, NLR: neutrophil-lymphocyte ratio, PT: prothrombin time, **APTT:** activated partial thromboplastin time, INR: international normalized ratio, **PCT:** procalcitonin, **AST:** aspartate aminotransferase, **LDH:** lactate dehydrogenase, **ALT:** alanine aminotransferase, **Bun:** blood urea nitrogen, **Cr:** creatinine, **CRP:** C reactive protein, **SII:** Systemic immune-inflammation index.

Obstetric, perinatal and clinical outcomes are presented in Table-II. Birthweight, APGAR scores and obstetric complication rates were similar between two groups p>0.005 for all. Hepatic injury was shown to be more frequent with advancing gestational week as shown in Table-II. Maternal complications related with severe disease such as pneumonia, system inflammatory response syndrome, sepsis, acute respiratory distress syndrome; multiple organ failure and disseminated intravascular coagulation were also more common in women with liver injury.

**Table-II:** Obstetric, perinatal and clinical outcomes.

	Control group n=63	Patient group n=33	P value
Pregnancy day on admission	196 42-271	224 140-281	0,009
Pregnancy trimester on admission	3 1-3	3 2-3	0,004
Labour status			0,424
-Vaginal	16	7	
-Ceserean	47	26	
Number of entubation day	0,2± 1,64	0,6±2,4	0,319
Number of intensive care day	0,4±1,9	1,1±3,0	0,039
Convalescent plasma level	3 4,8 %	7 21,2 %	0,015
CBAP	3 4,8 %	4 12,1 %	0,181
Nasal oxygen level	47 74,6 %	31 94,0 %	0,017
High oxygen	3 4,8 %	4 12,1 %	0,181
Entubation	2 3,2 %	4 12,1 %	0,104
Pneumonia	23 36,5 %	23 69,7 %	0,002
SIRS	24 38,1 %	24 72,7 %	0,001
Sepsis	1 1,6 %	4 12,1 %	0,046
ARDS	1 1,6 %	4 12,1 %	0,046
MOF	1 1,6 %	4 12,1 %	0,046
DIC	1 1,6 %	4 12,1 %	0,046
Birth weight, g	2973,6±674,6	2776,0±759,2	0,177
Apgar 1.min	8 7-10	8 8-9	0,227
Apgar 5. Min	10 8-10	10 9-10	0,283
Obstetric complication	2 3,2 %	6 18,2 %	0,018
Hospital day on admission	3 0-23	9 0-17	0,001
Time of birth day	264,0 201,0-275,0	259,0 168,0-280,0	0,047
Labor maturity			0,165
-Term	44	19	
-Premature	19	14	
PROM	1	2	0,271
PPROM	0	1	0,344
Preeclampsia	1	1	0,572
Placental abruption	1	0	0,656
Maternal death	1	4	0,046
Fetal death	0	1	0,344

**CBAP:** Continuous positive airway pressure, **SIRS:** systemic inflammatory response syndrome, **ARDS:** acute respiratory distress syndrome, **MOF:** multiorgan failure, **DIC:** Disseminated intravascular coagulation,** PROM:** Premature Rupture of Membranes, **PPROM:** Preterm premature rupture of membranes.

## DISCUSSION

This is one of the few studies that analyzes clinical and laboratory data of pregnant patients with SARS-CoV-2 with and without liver injury. In our study, the prevalence of liver injury in pregnant COVID-19 patients was found to be 34.4%. According to the results obtained from the Wuhan laboratory, it was shown that 23.8-44.4% of pregnant patients with COVID-19 had liver injury, and this was consistent with our finding.^20^ It has been reported that 45.7% of non-pregnant patients have liver injury, which is a higher frequency than observed in pregnant patients.^1^

Many studies have reported that hyperinflammation plays an important role in COVID-19 related deaths and in the pathogenesis of the disease. In our study, we found that inflammatory markers such as CRP, SII, NLR and IL-6 were higher in pregnant COVID-19 patients with liver injury.^21^ The course, complications, frequency, maternal and fetal effects of COVID-19 infection during pregnancy are still unknown. Immune dysregulation and high levels of proinflammatory cytokines that occur during the response to SARS-CoV-2 are the main causes of tissue damage. However, the pathophysiological mechanism of the disease is not fully known.^22^ In addition, it has been recently reported that elevated inflammatory markers are positively correlated with the severity of COVID-19.^15^

In this study, no significant difference was found between the groups in terms of obstetric complications except maternal death. During pregnancy, the immune system not only recognizes and fights infections, but also regulates unwanted immune responses by developing tolerance to its own antigens. According to Mor et al. anti-inflammatory phase in the second trimester of pregnancy may offer some protection against severe COVID-19.^23^ In this study, no significant difference was found between the groups in terms of laboratory and obstetric parameters in all three trimesters. Young and female patients are found to be more likely to have severe COVID-19 illness than older and male patients.^24^

Therefore, the low rates of severe COVID-19 in pregnant patients may be due to age and gender effects. How and why patients with liver injury have higher inflammation than those without is still a dilemma. In this study, 81.8% of 33 pregnant patients with liver injury gave birth to healthy babies, while this rate was 96.8% in those without liver injury. This may be evidence that liver damage worsens newborn outcomes.

Elevated liver enzymes in pregnant women can result from various causes aside from SARS-CoV-2 infection. One common factor is a condition known as intrahepatic cholestasis of pregnancy ICP, which occurs when there is a disruption in the flow of bile, leading to a buildup of bile acids in the liver. This condition can cause liver enzymes to rise and is associated with intense itching, particularly in the third trimester.^25^ Additionally, preeclampsia, a potentially serious pregnancy complication characterized by high blood pressure and damage to organs like the liver, can also lead to elevated liver enzymes.^26^ Other factors may include pre-existing liver conditions, such as viral hepatitis or fatty liver disease, which can become exacerbated during pregnancy due to hormonal changes and increased stress on the liver. Proper prenatal care and monitoring are crucial in identifying and managing these underlying causes to ensure the well-being of both the mother and the developing fetus.

It has been reported that the liver damage observed in COVID-19 patients may be directly related to the SARS-CoV-2 infection itself or to the drugs used in the treatment of SARS-CoV-2.^11^, ^27^ Since the cohort in this study consisted only of pregnant patients, no drugs that could cause liver injury were used. Therefore, the liver injury observed in all pregnant women is more likely to be caused by SARSCoV-2. Antiviral drugs used, including lopinavir and ritonavir, can also cause liver injury in COVID-19 patients as well as aggravate existing damage.^1^, ^14^ Further studies are needed to understand the mechanism of liver injury in COVID-19.

Liver damage in pregnant women with SARS-CoV-2 infection can indeed have distinct effects on perinatal outcomes compared to maternal outcomes. This differentiation is due to the complex interplay of factors involving the virus, the maternal immune response, and the physiology of pregnancy. The placenta acts as a barrier between the maternal and fetal circulations, providing some protection to the fetus. While SARS-CoV-2 can lead to liver damage in the mother, it does not necessarily directly affect the fetus through the placenta.^28^ Most newborns of mothers with SARS-CoV-2 infection do not show significant congenital abnormalities or developmental issues directly attributed to the virus. Perinatal outcomes are generally favorable in terms of infant health.^29^ Vertical transmission transmission of the virus from mother to baby during pregnancy is relatively rare, and even when it occurs, severe fetal or neonatal illness is uncommon.^29^ In summary, while liver damage itself may not directly impact perinatal outcomes, it can serve as a marker for more severe COVID-19 in pregnant women. The maternal immune response and the physiological changes associated with pregnancy play a crucial role in determining how the virus affects the mother and fetus differently. It is essential for pregnant individuals to receive timely medical care and close monitoring to manage any potential complications related to both liver damage and the SARS-CoV-2 infection during pregnancy.

The liver function of non-pregnant COVID-19 patients requiring intensive care has been reported to be significantly worse than those not in intensive care.^30^ Similar features were reported in an another study of 138 patients in Wuhan, China.^31^ There is widespread concern and evidence in these studies that it causes liver damage in Covid-19. Although the same concerns are valid for Covid-19 positive pregnant patients, there are two studies conducted on pregnant patients on this subject. To the best of our knowledge, this study will be the third on this topic in the literature.

### Limitations

It includes the small sample size and secondly, the lack of dynamic monitoring of liver function. A larger longitudinal cohort is needed to clarify the role of SARS-CoV-2 infection on liver injury. The strengths of this study are that it was conducted only in a pregnant cohort and it has an important reference value for improving care in this patient group.

## CONCLUSION

Our study showed that pregnant patients with liver injury had worse inflammation response than those without liver injury. All women with Covid-19 diagnosis should be tested in terms of liver injury. Women with elevated liver enzymes are tended to have a severe disease. Obstetric and perinatal outcomes were found to be similar between groups with and without liver injury. The impact of Covid-19 on pregnancy outcomes may be influenced by several factors including hepatic injury.

### Author’s contributions:

**NGK:** protocol/project development, data collection, data analysis, manuscript writing/editing.

**GO:** data collection, data analysis, manuscript writing/editing.

**FA:** data analysis, manuscript writing/editing.

**MK:** protocol/project development, data collection, data analysis, manuscript writing/editing and is responsible for the accuracy or completeness of the work.

All authors read and approved the final version of the manuscript.
